# Long-term treatment with active Aβ immunotherapy with CAD106 in mild Alzheimer’s disease

**DOI:** 10.1186/s13195-015-0108-3

**Published:** 2015-04-27

**Authors:** Martin R Farlow, Niels Andreasen, Marie-Emmanuelle Riviere, Igor Vostiar, Alessandra Vitaliti, Judit Sovago, Angelika Caputo, Bengt Winblad, Ana Graf

**Affiliations:** Department of Neurology, Indiana University School of Medicine, 355 West 16th Street, Suite 4700, Indianapolis, IN 46202 USA; Karolinska Institutet, Dept NVS, Center for Alzheimer Research, Division for Neurogeriatrics, Novum, Huddinge, SE-141 57 Stockholm Sweden; Karolinska University Hospital Huddinge, Geriatric Clinic, Clinical Trial Unit, SE-141 86 Stockholm, Sweden; Novartis Pharma AG, Basel, CH-4002 Switzerland

## Abstract

**Introduction:**

CAD106 is designed to stimulate amyloid-β (Aβ)-specific antibody responses while avoiding T-cell autoimmune responses. The CAD106 first-in-human study demonstrated a favorable safety profile and promising antibody response. We investigated long-term safety, tolerability and antibody response after repeated CAD106 injections.

**Methods:**

Two phase IIa, 52-week, multicenter, randomized, double-blind, placebo-controlled core studies (2201; 2202) and two 66-week open-label extension studies (2201E; 2202E) were conducted in patients with mild Alzheimer’s disease (AD) aged 40 to 85 years. Patients were randomized to receive 150μg CAD106 or placebo given as three subcutaneous (2201) or subcutaneous/intramuscular (2202) injections, followed by four injections (150 μg CAD106; subcutaneous, 2201E1; intramuscular, 2202E1). Our primary objective was to evaluate the safety and tolerability of repeated injections, including monitoring cerebral magnetic resonance imaging scans, adverse events (AEs) and serious AEs (SAEs). Further objectives were to assess Aβ-specific antibody response in serum and Aβ-specific T-cell response (core only). Comparable Aβ-immunoglobulin G (IgG) exposure across studies supported pooled immune response assessments.

**Results:**

Fifty-eight patients were randomized (CAD106, n = 47; placebo, n = 11). Baseline demographics and characteristics were balanced. Forty-five patients entered extension studies. AEs occurred in 74.5% of CAD106-treated patients versus 63.6% of placebo-treated patients (core), and 82.2% experienced AEs during extension studies. Most AEs were mild to moderate in severity, were not study medication-related and did not require discontinuation. SAEs occurred in 19.1% of CAD106-treated patients and 36.4% of placebo-treated patients (core). One patient (CAD106-treated; 2201) reported a possibly study drug-related SAE of intracerebral hemorrhage. Four patients met criteria for amyloid-related imaging abnormalities (ARIA) corresponding to microhemorrhages: one was CAD106-treated (2201), one placebo-treated (2202) and two open-label CAD106-treated. No ARIA corresponded to vasogenic edema. Two patients discontinued extension studies because of SAEs (rectal neoplasm and rapid AD progression, respectively). Thirty CAD106-treated patients (63.8%) were serological responders. Sustained Aβ-IgG titers and prolonged time to decline were observed in extensions versus core studies. Neither Aβ_1–6_ nor Aβ_1–42_ induced specific T-cell responses; however, positive control responses were consistently detected with the CAD106 carrier.

**Conclusions:**

No unexpected safety findings or Aβ-specific T-cell responses support the CAD106 favorable tolerability profile. Long-term treatment-induced Aβ-specific antibody titers and prolonged time to decline indicate antibody exposure may increase with additional injections. CAD106 may be a valuable therapeutic option in AD.

**Trial registration:**

ClinicalTrials.gov identifiers: NCT00733863, registered 8 August 2008; NCT00795418, registered 10 November 2008; NCT00956410, registered 10 August 2009; NCT01023685, registered 1 December 2009.

**Electronic supplementary material:**

The online version of this article (doi:10.1186/s13195-015-0108-3) contains supplementary material, which is available to authorized users.

## Introduction

Alzheimer’s disease (AD) is the most common cause of dementia in elderly populations, with an estimated worldwide prevalence of 35.6 million individuals in 2010, a number expected to almost quadruple by 2050 [1]. The global economic cost of the disease is huge and was estimated in 2010 to total US$604 billion [2]. Current management of AD involves symptomatic treatment with cholinesterase inhibitors (ChEIs), *N*-methyl-d-aspartate receptor partial antagonists and other drugs used to treat secondary behavioral symptoms associated with the illness [3,4].

Increased understanding of the pathogenesis of AD has identified a key role for amyloid-β (Aβ) peptide deposits in the development of AD [5]. Blocking aggregation of Aβ peptides and reducing Aβ plaque burden using disease-modifying immunotherapies is a promising approach in the treatment of AD [5-13]. However, recent phase III trial results of using monoclonal antibodies targeting Aβ have demonstrated equivocal evidence for efficacy in patients with mild-to-moderate AD [14,15], although clinical studies with solanezumab in patients with mild AD are continuing [14].

In a phase II trial of an active immunotherapy antibody to full-length Aβ peptide (AN1792 protein given with the adjuvant QS-21), researchers reported meningoencephalitis in a subset of patients, which led to concerns regarding T-cell activation [16]. In contrast, the novel, active immunotherapy CAD106 is targeted against a small peptide fragment of Aβ (Aβ_1–6_) and is conjugated to a carrier (Qβ bacteriophage). CAD106 is designed to ensure repetitive antigen (Aβ_1–6_) presentation and strong B-cell response, stimulating the generation of Aβ-specific antibodies while avoiding initiation of Aβ-specific T-cell responses [11].

Preclinical evidence suggests that CAD106 is capable of reducing Aβ accumulation in the brain by inducing antibodies that interfere with amyloid deposition and by binding to Aβ aggregates [11]. Following these positive preclinical findings [11], researchers in the first-in-human CAD106 study (CCAD106A2101; NCT00411580) investigated safety, tolerability and Aβ-specific antibody response following three subcutaneous (s.c.) injections of CAD106 or placebo in patients with mild-to-moderate AD [17]. The findings from that study suggested that three injections of CAD106 (50 or 150 μg) have a favorable safety profile and are capable of eliciting promising antibody responses without inducing autoimmune reactions [17]. In that study, total plasma Aβ concentration increased, whereas free Aβ in plasma decreased, indicating *in vivo* binding of antibodies to Aβ. Furthermore, CAD106-induced antibodies were capable of reacting with amyloid plaque cores and Aβ oligomers *ex vivo* [18].

**Figure 1:**
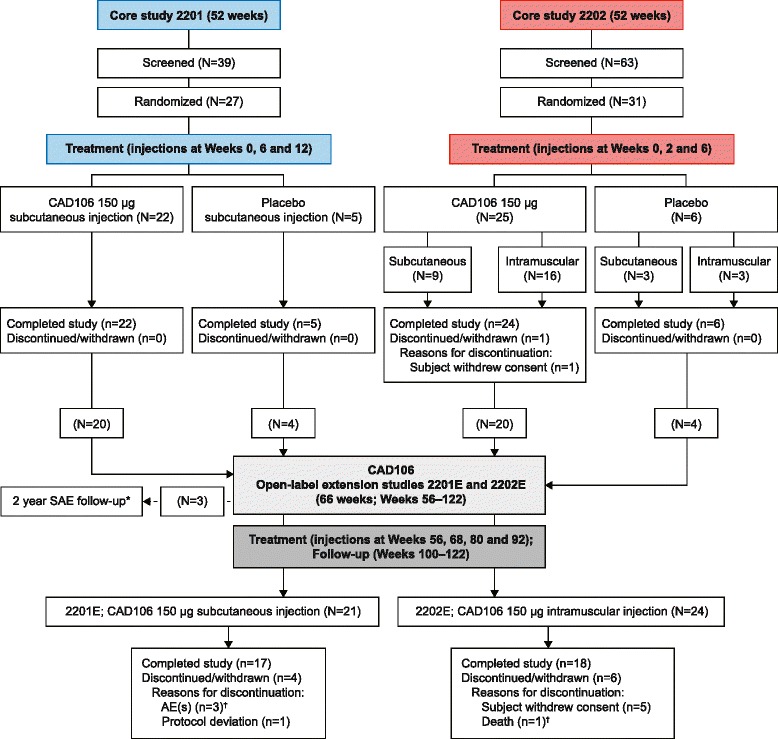
Overall study design: core and extension studies. *Not included in these analyses. ^†^Not suspected to be related to study drug. AE, Adverse event; N, number of patients in treatment group; n, Number of patients with a measurement; SAE, Serious adverse event.

We report the findings from two phase IIa, 52-week, multicenter, randomized, double-blind, placebo-controlled, parallel-group (core) studies of CAD106 and, following a 4-week rescreening period, their respective 66-week open-label extension studies (Figure 1). The objectives of these studies were to investigate safety and tolerability of repeated injections of 150μg CAD106 and evaluate antibody response following different dosing regimens in patients with mild AD dementia over a total duration of 122 weeks.

## Methods

Study protocols and amendments were reviewed by the independent ethics committee or institutional review board for each center (see Additional file 1). Studies were conducted according to the ethical principles of the Declaration of Helsinki.

### Patients

Patients were enrolled into the core studies in France, Sweden, Switzerland and the United Kingdom (CCAD106A2201; NCT00733863) and in the United States (CCAD106A2202; NCT00795418). Informed consent was obtained from each patient in writing before randomization. Inclusion and exclusion criteria for the core studies were identical. Patients were male or female (not of childbearing potential), aged 40 to 85 years and had a diagnosis of AD according to the *Diagnostic and Statistical Manual of Mental Disorders, Fourth Edition* [19] and probable AD according to the Work Group of the National Institute of Neurological and Communicative Disorders and Stroke and the Alzheimer’s Disease and Related Disorders Association [20]. At the time of study entry, patients were required to have mild AD (Mini Mental State Examination (MMSE) score 20 to 26), be untreated or on a stable dose (previous 6 weeks) of ChEI or other AD treatment, and to be in at least daily contact with a primary caregiver. Exclusion criteria included other medical or neurological conditions contributing significantly to the patient’s dementia; a history (past 2 years) or current diagnosis of central nervous system (CNS) inflammation indicative of meningoencephalitis or another immune disorder; a history of clinical stroke, intracranial hemorrhage or aneurysm; current diagnosis of significant cerebrovascular disease; and evidence of more than two cerebral microhemorrhages identified by a magnetic resonance imaging (MRI) central reader.

Patients who completed the core studies without major safety concerns were eligible for inclusion in the extension studies (CCAD106A2201E1, NCT00956410; and CCAD106A2202E1, NCT01023685). Patients who developed any condition listed in the core study exclusion criteria or who developed further conditions, such as neurodegenerative or psychiatric disorders, were excluded from the extension studies.

Patients required discontinuation from core or extension studies in the event of: development of two or more new brain microhemorrhages, meningoencephalitis or other severe immune reaction, treatment-emergent MRI findings of concern, moderate-to-severe symptomatic neurological abnormalities or unexpected worsening of dementia, or severe local or systemic reaction to the first or second injection.

Patient safety was monitored by an independent Data Monitoring Committee (DMC) who applied prespecified criteria listed in the protocols for suspension of immunization in specific individuals of concern or in all patients.

### Randomization and masking

Patients who met the core study eligibility criteria during the 3-week screening period were randomized (4:1; blocked by center) to receive CAD106 (150 μg) or placebo. The randomization list was generated at Novartis. (Basel, Switzerland) Identity of treatment was concealed for all study participants and personnel, with the exception of pharmacists handling study medication, members of the DMC, and the designated unblinded independent study team members.

### Study design and procedures

Dose selection and dosing schedule were based on the phase I study [17]. In study 2201, three injections were given s.c., at Weeks 0, 6 and 12. In study 2202, each patient received three injections either s.c. or by intramuscular (i.m.) route, at Weeks 0, 2 and 6. During the open-label extension studies, all patients received four injections of CAD106 150 μg at 12-week intervals (s.c. in 2201E1; i.m. in 2202E1) (Figure 1).

### Study objectives and outcomes

The primary objective was to evaluate the safety and tolerability of repeated injections of CAD106 150 μg in patients with mild AD over the course of 52 weeks and following a 66-week open-label extension. Further objectives included (1) determination of Aβ-specific antibody response to CAD106 (Aβ-immunoglobulin G (IgG) in serum and cerebrospinal fluid (CSF)) and increase in plasma Aβ_1–40_ from baseline, (2) characterization of Aβ-specific (Aβ_1–6_ and Aβ_1–42_) and Qβ-induced (as a positive control) T-cell responses in peripheral blood mononuclear cells (PBMCs; core studies only), (3) exploration of potential treatment effect on disease-related biomarkers in CSF and (4) exploration of potential treatment effects on disease progression as measured by volumetric cerebral MRI and clinical assessments.

### Assessments

All data were recorded on an electronic Case Report Form during core and extension studies.

Safety and tolerability outcomes were monitored throughout the studies and were assessed by performing general physical examinations, neurological examinations, 12-lead electrocardiography (ECG), vital sign measurements, laboratory evaluations in blood and CSF, cerebral MRIs and adverse event (AE) and serious AE (SAE) monitoring. Patients or their caregivers were instructed to record details of local and systemic injection-related reactions in a diary over a 7-day period following each injection. MRI was performed at screening, three times post-baseline during the core studies (Weeks 10, 20 and 52 (2201); Weeks 14, 26 and 52 (2202)) and three times during the extension studies (Weeks 74, 100 and 122). Lumbar punctures were scheduled at screening, at Weeks 20 (2201) and 14 (2202) and optionally at Weeks 96 and 122 (2201E and 2202E). Antibody titers and AD biomarkers were measured at those different CSF sampling time points. Antigen-specific T-cell assays (enzyme-linked immunospot) were performed on PBMC samples obtained at screening and 2 weeks after the second and third injections in the core studies only.

Cerebral MRI findings at screening and newly occurring findings were assessed by an independent central radiologist. Examinations were conducted using 1.5-T scanners, with harmonized setup across centers, to detect white matter lesions, microhemorrhages (amyloid-related imaging abnormalities (ARIA) corresponding to microhemorrhages (ARIA-H)), edema and inflammation, and included the following image sequences: fluid-attenuated inversion recovery (FLAIR; ARIA corresponding to vasogenic edema (ARIA-E)); T2-weighted scans, T2*-weighted gradient echo scans (microhemorrhages), diffusion-weighted imaging (to diagnose vascular pathology and differentiate between vasogenic and cytotoxic edema) and T1-weighted scans (volumetric MRI).

Aβ-specific antibody titers in serum were measured using an enzyme-linked immunosorbent assay at each scheduled visit from screening until the end of the study, as described previously [21]. Antibody titers were expressed in units in reference to rhesus monkey reference serum to allow comparison across CAD106 studies. Total plasma Aβ_1–40_ (free and bound) levels were measured using a Meso Scale Discovery (MSD)-based assay (catalog number K150FTE-3; Meso Scale Diagnostics, Rockville, MD, USA) and were compared with baseline levels. This assay utilizes combination capture with anti-Aβ_1–40_ targeted at the N-terminal domain of Aβ_1–40_ and ruthenium-labeled detection monoclonal antibody (4G8). The assay was validated and adapted for semiquantification of longitudinal changes in Aβ_1–40_ in human plasma only. Additional analyses were performed on selected patients’ samples to determine Aβ-IgG affinity maturation during core study 2201.

Aβ- and Qβ-induced T-cell responses were assessed based on interferon-gamma (IFN-γ) cytokine. Antigen-specific T-cell response was determined by the number of cells secreting IFN-γ, comparing cell number from non-stimulated wells (C; in medium) with cell number from antigen-stimulated wells (R). Mean number of cells (standard deviation (SD)) was based on three replicate experiments. Antigen-specific T-cell response was derived at each visit as ((mean (R) − SD (R)) − (mean (C) + SD (C))), and mean change (SD) from baseline in T-cell response was assessed at each visit.

Disease-related biomarkers in CSF comprised Aβ_1–40_ and Aβ_1–42_, total tau and hyperphosphorylated tau (phospho-tau). An MSD-based sandwich assay was used to measure the concentration of Aβ_1–40_ and Aβ_1–42_. Concentration of total tau and phospho-tau_181_ were measured by solid-phase enzyme immunoassay using kits provided by Fujirebio Europe (Gent, Belgium).

The effect of CAD106 on brain volume (measured in cm^3^) was calculated using a T1-weighted MRI scan, performed as part of the safety cerebral MRI. T1-weighted images were analyzed under supervision of the Novartis Clinical Imaging Unit using NeuroQuant software (CorTechs Labs, La Jolla, CA, USA) to identify and compute the volume of whole brain and its substructures (hippocampus, cerebral cortex, white matter and ventricles). The clinical assessments MMSE, Global Deterioration Scale, Alzheimer’s Disease Assessment Scale–Cognitive subscale (ADAS-Cog; 11 items), Trail Making Test Part A and category fluency were conducted by qualified personnel not involved in safety assessments. Caregiver completion of the Alzheimer’s Disease Cooperative Study–Activities of Daily Living and Neuropsychiatric Inventory Questionnaire was performed under guidance of a study nurse or neuropsychologist.

### Statistical analysis

Sample size for the core studies was calculated based on the likelihood of detecting a 6% event rate for meningoencephalitis, as seen in the AN1792 study [16]. Twenty-four patients on active treatment was considered sufficient; the probability of observing at least one case of meningoencephalitis with a true incidence rate of 6% was calculated to be 77.3%.

A pooled approach combining the two studies and their respective extensions, with up to seven injections in total through 122 weeks, was planned to enhance the chances of detecting any signal on safety outcome measures. The two studies were designed in parallel with similar features, with the exception of schedule of injections in the core studies and the route of administration. As the actual exposure to Aβ-IgGs was found to be comparable in the two studies (see Results), this pooled approach was used.

Analyses were based on pooled study data unless otherwise stated. All patients who received at least one injection and had at least one post-injection safety assessment were included in the pooled safety analysis set. The analyses presented here are based on this analysis set. For immune response and pharmacodynamic assessments, reports focus on patients in the pooled safety analysis set who were randomized to CAD106 in the core studies (that is, received CAD106 throughout the core and extension studies). Descriptive group comparisons were predominantly of patients who received CAD106 versus placebo during the core studies.

Patients treated with CAD106 were classified as serological responders or non-responders based on serum Aβ-IgG titers during the core studies. The lower limit of quantification (LLOQ) of serum Aβ-IgG titers was determined as 8.93 U based on assay variability and baseline titers observed in the phase I CAD106 study [17]. The responder threshold was increased after learnings from the 2201E and 2202E extension studies were implemented. Serological responders were defined as demonstrating a consistent antibody response after the second and third injections: Aβ-IgG titers above 16 U between the second and third injections and above 26.8 U after the third injection. The threshold of 16 U corresponds to the LLOQ plus three times the SD, and the threshold of 26.8 U corresponds to three times the LLOQ. Antibody response was characterized by the area under the titer curve (AUC), calculated using the trapezoidal method for observed data.

Change from baseline in phospho-tau in CSF was analyzed by using a mixed-effects repeated measurement model, adjusted for phospho-tau baseline value and including visit week and the interaction as repeated factors.

## Results

### Patients

Fifty-eight patients were randomized and exposed to study drug during the core studies: 47 to CAD106 and 11 to placebo. Forty-five patients entered the extension studies and received open-label treatment (CAD106). Details of patient flow and disposition through the studies are shown in Figure 1. All but two patients received three scheduled injections during the core studies. In the extension studies, all patients received at least two injections of CAD106; 19 patients (90.5%) in 2201E received all four injections. In 2202E, only 13 patients (54.2%) received the scheduled four injections. Eight patients (33.4%) did not receive the fourth injection, owing to temporary suspension of CAD106 dosing during the evaluation of an SAE (subdural hematoma followed by intracerebral hemorrhage (ICH)), initially suspected to be related to study drug in another study with CAD106.

Baseline (core study) demographics and disease characteristics were generally well balanced between patients randomized to receive CAD106 versus placebo, although patients in the CAD106 pooled group were, on average, 2 years older and less frequently apolipoprotein E4 allele (ApoE4) carriers (60% versus 82%). There were no notable differences in MMSE scores between groups, with mean scores of approximately 22 (Table 1).

**Table 1:** **Patient demographics and core baseline disease characteristics by treatment (pooled safety analysis set)**

	**CAD106 (2201)**	**CAD106 (2202)**	**CAD106 (total)**	**Placebo (total)**
	**N = 22**	**N = 25**	**N = 47**	**N = 11**
Sex, n (%)				
Male	14 (63.6)	15 (60.0)	29 (61.7)	6 (54.5)
Female	8 (36.4)	10 (40.0)	18 (38.3)	5 (45.5)
Age (yr)				
Mean (SD)	65.6 (7.1)	70.5 (8.7)	68.2 (8.2)	66.3 (6.9)
Age group, n (%)				
<65 yr	10 (45.5)	6 (24.0)	16 (34.0)	6 (54.5)
65–75 yr	10 (45.5)	9 (36.0)	19 (40.4)	4 (36.4)
>75 yr	2 (9.1)	10 (40.0)	12 (25.5)	1 (9.1)
Race, n (%)				
Caucasian	22 (100.0)	24 (96.0)	46 (97.9)	10 (90.9)
Black	0 (0.0)	1 (4.0)	1 (2.1)	0 (0.0)
Other	0 (0.0)	0 (0.0)	0 (0.0)	1 (9.1)
Years of education				
Mean (SD)	11.7 (2.7)	14.2 (3.2)	13.1 (3.2)	12.2 (4.1)
MMSE score at baseline				
Mean (SD)	23.1 (2.2)	21.9 (1.9)	22.5 (2.1)	22.8 (2.3)
Time since first symptom of AD was noticed by patient/caregiver (yr)				
Mean (SD)	5.4 (2.7)	3.3 (2.2)	4.3 (2.6)	4.5 (1.8)
Time since first symptom of AD was first diagnosed by physician (yr)				
Mean (SD)	2.0 (1.7)	1.3 (1.3)	1.6 (1.5)	1.8 (1.3)
ApoE4 carrier status, n (%)*				
Missing	2 (9.1)	2 (8.0)	4 (8.5)	1 (9.1)
Non-ApoE4	9 (40.9)	3 (12.0)	12 (25.5)	1 (9.1)
One ApoE4	8 (36.4)	15 (60.0)	23 (48.9)	5 (45.5)
Two ApoE4	3 (13.6)	5 (20.0)	8 (17.0)	4 (36.4)

AD, Alzheimer’s disease; ApoE4, Apolipoprotein E4 allele; MMSE, Mini Mental State Examination; N, Number of subjects in treatment group; n, Number of patients with a measurement; SD, Standard deviation. *Percentage based on the number of patients genotyped.

### Safety and tolerability

During the core studies, AEs occurred in 35 patients (74.5%) treated with CAD106 and seven patients (63.6%) who received placebo. In the extension studies, the majority of patients reported at least one AE over the 66-week duration, although no unexpected safety findings were noted. Across the studies, most AEs were considered mild or moderate in severity, were not related to study drug and did not require study drug interruption. There were no differences in the severity of AEs or causality between groups.

The most frequently observed AEs in patients treated with CAD106 during the core studies were spread across multiple system organ classes; no specific pattern for incidence of AEs was detected (Table 2). The main categories of AEs with an increased incidence with CAD106 versus placebo included nervous system disorders (21.3%; the most frequent AE being headache (12.8%)) and general disorders and administration site conditions (29.8%; the most frequent AE being pyrexia (8.5%)).

**Table 2:** **Summary of deaths, serious adverse events, discontinuations due to serious adverse events, and adverse events, regardless of study drug relationship (core and extension studies; pooled safety analysis set)**

		**Core studies**				**Extension studies**
		**CAD106 (2201)**	**CAD106 (2202)**	**CAD106 (pooled)**	**Placebo (pooled)**	**CAD106 total (pooled)**
		**N = 22 (all s.c.)**	**N = 25 (n = 9 s.c.; n = 16 i.m.)**	**N = 47**	**N = 11**	**N = 45**
**Deaths**		**0 (0.0)**	**0 (0.0)**	**0 (0.0)**	**0 (0.0)**	**1 (2.2)**
**SAEs**		**4 (18.2)**	**5 (20.0)**	**9 (19.1)**	**4 (36.4)**	**6 (13.3)**
	Intracerebral hemorrhage	1 (4.5)**	0 (0.0)	1 (2.1)	0 (0.0)	0 (0.0)
**Discontinuations due to (S)AEs**		**0 (0.0)**	**0 (0.0)**	**0 (0.0)**	**0 (0.0)**	**2 (4.4)**
Amyloid-related imaging abnormalities*		**1 (4.5)**	**0 (0.0)**	**1 (2.1)**	**1 (9.1)**	**2 (4.4)**
	With hemorrhage	1 (4.5)**	0 (0.0)	1 (2.1)	1 (9.1)	2 (4.4)
**Any primary system organ class, n (%)**		**15 (68.2)**	**20 (80.0)**	**35 (74.5)**	**7 (63.6)**	**37 (82.2)**
Cardiac disorders		3 (13.6)	3 (12.0)	6 (12.8)	0 (0.0)	7 (15.6)
Gastrointestinal disorders		6 (27.3)	2 (8.0)	8 (17.0)	2 (18.2)	15 (33.3)
	Diarrhea	3 (13.6)	0 (0.0)	3 (6.4)	0 (0.0)	6 (13.3)
General disorders and administration site conditions		6 (27.3)	8 (32.0)	14 (29.8)	2 (18.2)	9 (20.0)
	Pyrexia	1 (4.5)	3 (12.0)	4 (8.5)	1 (9.1)	0 (0.0)
	Fatigue	1 (4.5)	2 (8.0)	3 (6.4)	1 (9.1)	6 (13.3)
Infections and infestations		8 (36.4)	3 (12.0)	11 (23.4)	3 (27.3)	16 (35.6)
	Nasopharyngitis	2 (9.1)	0 (0.0)	2 (4.3)	0 (0.0)	6 (13.3)
Injury, poisoning and procedural complications		0 (0.0)	5 (20.0)	5 (10.6)	2 (18.2)	9 (20.0)
Fall		0 (0.0)	3 (12.0)	3 (6.4)	1 (9.1)	3 (6.7)
Investigations		4 (18.2)	2 (8.0)	6 (12.8)	2 (18.2)	4 (8.9)
Musculoskeletal and connective tissue disorders		6 (27.3)	3 (12.0)	9 (19.1)	1 (9.1)	9 (20.0)
	Back pain	1 (4.5)	3 (12.0)	4 (8.5)	0 (0.0)	3 (6.7)
Nervous system disorders		4 (18.2)	6 (24.0)	10 (21.3)	2 (18.2)	11 (24.4)
	Headache	2 (9.1)	4 (16.0)	6 (12.8)	0 (0.0)	6 (13.3)
Psychiatric disorders		2 (9.1)	6 (24.0)	8 (17.0)	4 (36.4)	15 (33.3)
	Confusional state	0 (0.0)	3 (12.0)	3 (6.4)	0 (0.0)	3 (6.7)
	Depression	1 (4.5)	2 (8.0)	3 (6.4)	0 (0.0)	6 (13.3)
Renal and urinary disorders		2 (9.1)	4 (16.0)	6 (12.8)	1 (9.1)	4 (8.9)
Skin and subcutaneous tissue disorders		0 (0.0)	6 (24.0)	6 (12.8)	1 (9.1)	7 (15.6)

AE, Adverse event; ARIA-E, Amyloid-related imaging abnormalities corresponding to vasogenic edema; ARIA-H, Amyloid-related imaging abnormalities corresponding to microhemorrhage; FLAIR, Fluid-attenuated inversion recovery; ICH, Intracerebral hemorrhage; i.m., Intramuscular; N, Number of patients in treatment group; n, Number of patients reporting event; SAE, Serious adverse event; s.c., Subcutaneous; SOC, System organ class. *ARIA-H include two or more new microhemorrhages, subarachnoid hemorrhages or hemosiderosis. No ARIA-E findings were reported. One patient with T2-weighted FLAIR at Week 52 was confirmed as ischemic stroke at an unscheduled scan (Week 56 extension baseline), ruling out the likelihood of vasogenic edema. **The same patient experienced subarachnoid hemorrhage at Week 20 and ICH at Week 36. SOCs are presented in alphabetical order for incidences >10% in any CAD106 treatment group. Within each SOC, preferred terms for AEs with an incidence >10% in any CAD106 treatment group are shown. A subject with multiple occurrences of an AE under one treatment is counted only once in each AE category for that treatment. A subject with multiple AEs within a SOC or preferred term is counted only once in the total row for the core and extension studies. Ongoing events at the end of the core study are counted again in the extension studies; therefore, patients may be counted twice in the total columns for the core and extension studies.

Imbalance for cardiac disorders was observed during the core studies, with AEs reported in six patients (12.8%) who received CAD106 versus no patients who received placebo. In the extension studies, seven patients (15.6%) reported cardiac disorders; four of these patients had previously reported such events during the core studies. Of the nine patients with cardiac disorders, two patients required hospitalization: one for myocardial ischemia along with an AE of coronary artery stenosis, and one for complete atrioventricular block. In addition, four patients reported rhythm disturbances (bradycardia (n = 2), ventricular extrasystoles (n = 1), tachycardia (n = 1)), two reported cardiac perfusion-related AEs (angina pectoris, arteriosclerosis coronary artery) and one experienced left ventricular hypertrophy.

There were no incidences of meningoencephalitis, autoimmune disease or CNS inflammation, as confirmed by CSF laboratory assessments and regular MRI scans. No relevant findings were reported on ECGs, vital signs measurements or standard laboratory evaluations in patients treated with CAD106 or placebo. There was no increase in white blood cell count in CSF and no abnormalities in albumin or total IgG CSF : blood indices. Increase in oligoclonal bands in CSF only, with no concomitant signs or symptoms, was detected in two (9.1%) CAD106-treated patients during core study 2201.

Across the two core and extension studies, one patient died during the open-label extension study 2202E. The death was not considered to be related to study medication (progression of chronic obstructive pulmonary disease). There was no imbalance of SAEs during the core studies across treatment groups. Of the 13 reported SAEs, a single case of ICH was assessed by the investigator as potentially related to CAD106 during core study 2201. After receiving the three scheduled injections of CAD106, the patient did not meet the responder criteria and had last detectable Aβ-IgG at week 16 (12.6 U). A small sulcal subarachnoid hemorrhage was identified on the scheduled MRI at week 20, and an ICH was diagnosed during an unscheduled MRI at week 36, conducted after the patient experienced word-finding difficulties and disorientation. A relationship to study drug was considered possible by the investigator. The patient completed the core study safety monitoring until Week 52, but was not eligible to continue in the extension.

During the extension studies, two patients discontinued as a result of SAEs (SAEs of rectal neoplasm (2201E) and rapid AD progression (2202E)).

During the core studies, two patients presented with findings meeting ARIA-H criteria: one placebo-treated patient (9.1%) presented with two or more new microhemorrhages (2202), and one CAD106-treated patient (2.1%) presented with subarachnoid hemorrhage (2201; the same patient who reported SAE of ICH). During the extension studies, two further patients (4.3%) presented with two or more new microhemorrhages. All four patients across the studies remained asymptomatic. The three CAD106-treated patients were carriers of one ApoE4 allele, whereas the placebo-treated patient was a non-carrier. Only one of the CAD106-treated patients (with a finding of at least two new microhemorrhages during the extension) met the criteria for being a serological responder.

No MRI findings compatible with ARIA-E criteria were reported. One asymptomatic case of new hyperintense T2-weighted lesion (CAD106-treated patient (2202)) at Week 52 was confirmed as related to a small cortical infarction (stroke) upon repeat MRI.

**Figure 2:**
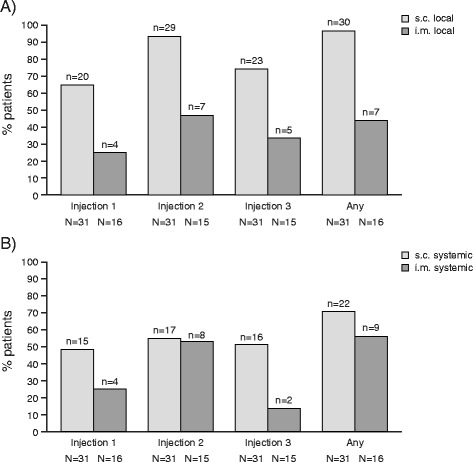
**Incidence of injection-related reactions in CAD106-treated patients by injection and route (subcutaneous (2201 and 2202) versus intramuscular (2202)). (A)** Local reactions. **(B)** Systemic reactions. Injection-related reactions were collected via a patient diary over the 7 days following each injection. Injection 1 at Week 0 (2201 and 2202), injection 2 at Week 6 (2201) or Week 2 (2202), injection 3 at Week 12 (2201) or Week 6 (2202). Local reactions: bruising (ecchymosis), redness, induration, swelling, local pain. Systemic reactions: fever, chills, malaise, muscle pain (myalgia), joint pain (arthralgia), headache, sweating, fatigue. i.m., Intramuscular; s.c., Subcutaneous.

Brief, self-limited injection-related reactions, either local reactions (for example, redness, induration or swelling) or systemic reactions (such as flu-like symptoms, fever, headache, fatigue, chills or muscular pain), were observed in the majority of patients receiving CAD106. The reactions were mostly mild to moderate and did not require treatment. None of the reactions led to discontinuation of injections. In one case, a patient experienced transient systemic reactions (syncope) associated with the second and third injections in the core study (2202; s.c. route) that were suspected to be related to study drug. This patient entered the extension study and received the fourth injection (first injection of extension) under supervision at the hospital. All four injections in the extension study were well tolerated, and there were no further reports of similar systemic reactions in this patient. During the core studies, tolerability with the i.m. route of injection (2202) was comparable with the s.c. route (2201 and part of study 2202) (Figure 2). The i.m. route of injection was found to result in fewer local reactions across all injections and in fewer systemic reactions with the third injection.

### Antibody response

**Figure 3:**
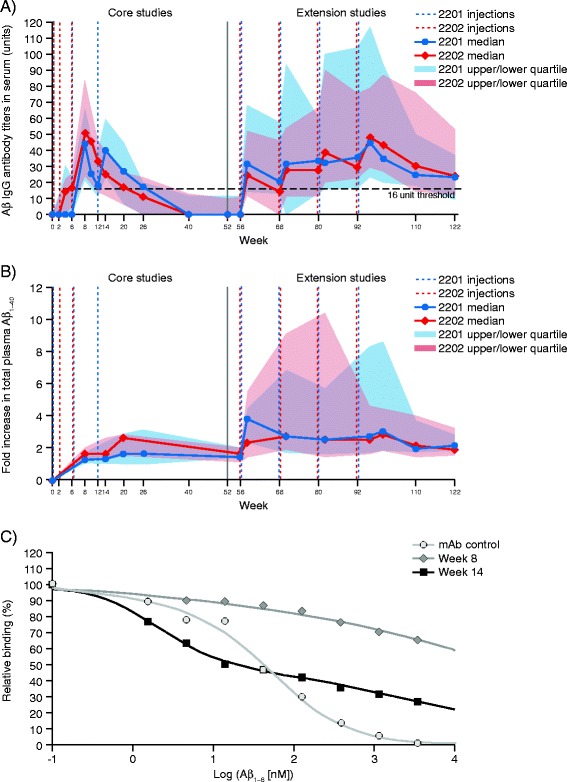
**Amyloid-β-specific antibody response. (A)** Median and interquartile range (IQR) Q1–Q3 Aβ-IgG titers (units) in serum by study and visit (pooled safety analysis set).* **(B)** Median and IQR Q1–3 fold increase in Aβ_1–40_ in plasma,^†^ by study and visit (pooled safety analysis set).* **(C)** Mean Aβ-IgG affinity maturation between second and third injections compared with similar N-terminal monoclonal antibody control. *Only patients who received CAD106 in the core studies and entered the extension studies are included. At each time point, only patients with a value at baseline and that time point are included. ^†^Values below the LLOQ (LLOQ = 8.93) were set to 67 pg/ml. Binding of Aβ_1–6_ to the serum samples containing either a reference antibody or to samples from one representative serological responder to CAD106 from study 2201 at Week 8 (2 weeks after the second injection, Aβ-IgG titers = 48.5 U) and from the same patient at Week 14 (2 weeks after the third injection, Aβ-IgG titers = 278 U). Aβ, Amyloid-β; IgG, Immunoglobulin G; IQR, Interquartile range; LLOQ, Lower limit of quantification; mAb, Monoclonal antibody.

No patient presented with serum Aβ-IgG titers above the LLOQ at baseline. Patients randomized to placebo did not develop Aβ-IgG titers during the core studies. The antibody response characteristics up to Week 20 (AUC and peak value in serum) were similar across the core studies, but showed high variability between individual patients (Figure 3A). In core study 2202, patients received CAD106 via s.c. or i.m. route of administration. Patients who received i.m. injections generally showed a higher response (n = 15, mean AUC = 11,107.13 U/day) than those who received CAD106 by s.c. administration (n = 9, mean AUC = 4,743.5 U/day). No difference was observed between s.c. (2201E1) versus i.m. (2202E1) administration routes in the extension studies.

Sustained Aβ-IgG titers above the serological responder threshold were observed between consecutive injections in the extension studies, with almost 50% of patients maintaining Aβ-IgG titers above the threshold at the end of the study (30 weeks after last injection of CAD106; median 23.4 U and 24.0 U for 2201E and 2202E, respectively). A prolonged time to decline in antibody titers was detected in the extension studies compared with the core studies for patients who received CAD106 during the core and extension studies. Of those patients treated with CAD106 during the core studies, 30 (63.8%) were considered serological responders. Placebo patients who continued in the extension and received open-label treatment with CAD106 reached similar Aβ-IgG titers as observed in CAD106-treated patients during the core studies (data not shown).

The total concentration of plasma Aβ_1–40_ (free and bound fractions) in the periphery was expected to increase upon binding to Aβ-IgGs. As shown in Figure 3B, total plasma Aβ_1–40_ increased with repeated injections of CAD106. In the extension studies, the range of plasma Aβ_1–40_ concentrations was much larger than in the core studies, with the upper quartile of plasma Aβ_1–40_ distribution reaching a tenfold increase compared with baseline concentrations. At the same time points, the upper quartile of Aβ-IgG titers increased by approximately twofold. No increase in plasma Aβ_1–40_ was seen in placebo-treated patients. The concentration of plasma Aβ_1–40_ correlated with the AUC of Aβ-IgGs, with Spearman’s correlation coefficients of 0.70 after the third injection (Week 20 for study 2201; Week 14 for study 2202) and 0.60 after the seventh injection (Week 96 of both studies).

The apparent maturation of affinity of CAD106-generated Aβ-specific antibodies was determined in two samples from study 2201, collected at Week 8 (two weeks after second injection) and Week 14 (two weeks after third injection), respectively. Representative patients showing a serological response were selected, and their samples were compared with an Aβ_1–6_ monoclonal antibody control. The affinity profiles demonstrated wide polyclonal distribution of Aβ-specific antibody response, with a notable improvement in overall apparent affinity of response between Weeks 8 and 14, with a subpopulation of antibodies reaching nanomolar affinity (*K*_d_) for Aβ_1–6_ at Week 14 (Figure 3C).

At each lumbar puncture, all patients who contributed CSF samples were also tested for Aβ-IgGs in CSF. Only two of the 47 CAD106-treated patients had quantifiable Aβ-IgGs in CSF; both patients had high serum Aβ-IgGs at the same time points. One patient had CSF IgG titer of 2.87 U and serum level of 1,240 U (2201E1 at Week 96), and the other had a CSF IgG titer of 1.13 U and serum level of 260 U (2202 at Week 14). These titers yield a CSF : serum ratio of antibodies close to 0.2%.

### T-cell response

A Qβ-induced T-cell response (change from baseline in IFN-γ-secreting cell numbers) was consistently detected in patients treated with CAD106. Mean change from baseline (SD) in Qβ-induced T-cell response increased with injections and reached a maximum 2 weeks after the third injection in both studies (Week 14 (2201) = 31.0 (48.95) and Week 8 (2202) = 39.2 (67.34), respectively). No change was observed at any time point with Aβ_1–6_ or Aβ_1–42_ in patients treated with placebo. Neither Aβ_1–6_ nor Aβ_1–42_ induced any specific T-cell responses. The number of T cells secreting IFN-γ remained stable up to 2 weeks after the third injection. Induction levels were −7.5 (32.17) and 0.6 (5.98) at Week 14 (2201) and −0.5 (0.87) and 0.0 (0.89) at Week 8 (2202) for Aβ_1–42_ and Aβ_1–6_, respectively.

### Pharmacodynamic results

In general, concentrations of disease-related biomarkers were comparable between treatment groups at core study baseline. Overall in the core studies, no treatment effect was observed on any CSF disease-related markers with CAD106 versus placebo at the early CSF time point of 8 weeks after the third injection (Week 20 (2201) and Week 14 (2202)). During the extension studies, lumbar puncture was optional and only a few patients contributed CSF (13 at Week 96 and 8 at Week 122 on open-label treatment with CAD106). Decreases in CSF phospho-tau concentrations from core study baseline were observed in the extension studies. Baseline values at the start of CAD106 open-label treatment (Week 56) were not available for comparison. For patients treated with CAD106 throughout the core and extension studies, the model base (mixed-effects repeated-measures analysis) indicated consistent results between studies. The least squares mean change over time was −24.38 pg/ml (95% confidence interval (CI): −28.25; −20.51) at Week 96 and −21.29 pg/ml (95% CI: −26.42; −16.16) at Week 122. Of note, the two patients who received placebo during the core studies also showed a decrease in phospho-tau from core study baseline when receiving CAD106 in the extension studies.

Brain atrophy consistent with natural disease progression, and in line with published annual rates [22], was observed across all studies and treatment groups. There were no treatment-related findings with respect to total brain volume or total ventricle volume.

There were no treatment-related benefits with respect to outcomes on clinical scales compared with placebo at any time point. Patients generally demonstrated deterioration on all assessment scales over the course of the studies, consistent with the progressive nature of AD. Of those patients who continued in the extension studies, mean (SD) change from baseline on the ADAS-Cog at Week 52 was 5.6 (7.2) for CAD106 versus 5.0 (7.3) for placebo; at Week 122 (all patients receiving CAD106), the mean change from baseline was 12.8 (11.1).

## Discussion

The results of these studies suggest that long-term treatment with CAD106 is in line with the findings from the phase I study and demonstrate that CAD106 is capable of generating a consistent antibody response over the course of seven injections [17].

The study population was representative of patients with mild AD. In a review of ApoE status and its relationship to AD, the frequency of ApoE4 carriers was estimated to be 58% in the AD population versus 23% in the general population [23]. The overall proportion of ApoE4 carriers in these studies (>60%) is relevant when evaluating the safety of CAD106 in ApoE4 carriers, who have been reported to be at greater risk for ARIA-E following treatment with the passive amyloid immunotherapy bapineuzumab [23]. Throughout the studies reported here, no patient met the criteria for ARIA-E and four patients met the criteria for ARIA-H. One patient who received CAD106 during the core study experienced a subarachnoid hemorrhage and was diagnosed with an ICH 16 weeks later, approximately 5 months after receiving the last injection of CAD106.

Our findings support the safety and tolerability of CAD106 observed in the phase I study [17]. There were no unexpected safety findings related to treatment with CAD106 and no occurrences of meningoencephalitis, autoimmune disease, CNS inflammation or asymptomatic vasogenic edema, in contrast to previous reports with Aβ antibody therapies [18]. Although events related to cardiac disorders were reported more frequently in CAD106-treated patients versus placebo during the core studies, a similar number of patients reported cardiac events in the CAD106 open-label extension studies. No specific pattern of disorder was identified, and the occurrence rate (<16% overall) was in line with that expected in an elderly population followed for up to 122 weeks.

The favorable tolerability and serological response of i.m. versus s.c. administration seen in core study 2202 resulted in selection of i.m. administration for future trials of CAD106.

Long-term administration of CAD106 was associated with a safety and tolerability profile similar to the initial injections received during the core studies. CAD106 was generally well tolerated, with most AEs considered mild or moderate in severity and consistent with that expected in an elderly population. They were generally not considered related to study medication, with the exception of short-lasting and self-limited injection-related reactions, which were experienced by most patients.

As observed in the phase I study [17], treatment with CAD106 during the core studies induced an antibody response against Aβ in the majority of patients. This finding provides supporting evidence that CAD106 150 μg is an effective dose for producing an antibody response. Antibody titers at Week 8 (after second injection in 2201 or third injection in 2202) were similar across the studies, indicating that the additional early injection at Week 2 in 2202 did not contribute to the magnitude of the immune response. Only two patients had a quantifiable level of Aβ-specific IgGs in CSF, each at one time point during the studies. The CSF : serum IgG ratio was in line with the expected ratio for diffusion of large molecules, such as antibodies, through the blood–brain barrier [24]. A full mechanism supporting the potential for peripheral antibodies targeting CNS Aβ has not yet been elucidated. Despite the small levels of Aβ-specific IgGs anticipated to reach the CSF, these antibodies can exert a multitude of effects on CNS Aβ [12,25].

Long-term treatment with CAD106 induced Aβ-specific antibody titers similar to the initial response, but with a prolonged time to decline, suggesting that antibody exposure may be increased between injections. The observed greater increase in total plasma Aβ_1–40_ with long-term treatment could be explained by affinity maturation of the Aβ-specific antibodies following repeated injections. Indeed, affinity maturation profiles of the antibodies for Aβ_1–6_ demonstrated a pronounced increase in affinity after the third CAD106 injection, compared with after the second injection in the same patient. However, the limited sample size means that these results should be interpreted with caution.

The absence of Aβ-specific T-cell responses in CAD106-treated patients suggests that CAD106 does not induce a T-cell-mediated immune response against Aβ, consistent with previous findings from the phase I study [17]. This mechanism was involved in the autoimmune meningoencephalitis observed with the full-length Aβ protein, AN1792 [16]. Positive control T-cell activation by the Qβ CAD106 carrier particle was shown in preclinical studies with CAD106 [11], but our findings represent the first time this response has been observed in humans. Overall, the T-cell response to CAD106 treatment supports the favorable tolerability profile of CAD106, minimizing the likelihood of autoimmune reactions while indicating its validity as an active immunization.

In addition to Aβ accumulation, AD pathology is characterized by neurofibrillary tangles formed of hyperphosphorylated tau protein, which has been hypothesized to be a downstream product of Aβ toxicity [26]. In the present study, phospho-tau concentrations in the extensions were numerically lower than in the core studies, irrespective of core study treatment. Although the relevance of these observations is difficult to assess in the absence of appropriate reference measurements, the apparent decrease in phospho-tau may be due to natural disease progression or a direct treatment effect after more than 2 years of disease evolution in patients with mild AD. Indeed, recent longitudinal data have demonstrated that CSF biomarkers, including phospho-tau, decrease in the later stages of AD [27,28].

No treatment-related differences were observed in brain volume or in any of the AD clinical scale measurements. However, the results on the clinical scales should be interpreted with caution, owing to small population sizes. Continuous exposure to antibody titers through regular injections, along with targeting earlier disease stages, might increase the likelihood of positive effects on clinical outcomes [17,29]. Indeed, recently published results modeling Aβ accumulation using amyloid positron emission tomography imaging demonstrated a sigmoidal pattern of amyloid accumulation within the brain and suggested that therapeutic strategies designed to reduce amyloid deposition may be most effective if administered early in the disease course [30,31]. A recent review summarized treatment development in AD over the last 30 years and highlighted the need for further detailed proof-of-concept studies with anti-amyloid therapies [32].

## Conclusions

The results of these studies indicate that long-term treatment with CAD106 induced antibodies to Aβ and increased Aβ_1–40_ levels in serum, suggesting that this approach can achieve target engagement in the periphery. The favorable safety profile of CAD106 observed in these studies supports active immunization with Aβ_1–6_ and, combined with the absence of a specific T-cell response against Aβ and the ability to produce a sustained antibody response, suggests that CAD106 may be a valuable therapeutic option in the chronic treatment of AD. A 12-week interval between CAD106 injections is currently being evaluated across seven injections. This regimen is expected to result in continuous exposure to higher antibody titers. Our data support the continued investigation of active immunization strategies in AD and offer promise for immunotherapy as a future treatment option.

## Additional file

Additional file 1:
**List of independent ethics committees or institutional review boards.** Independent ethics committee and institutional review board names and addresses for each study and each participating study center are given.

## Abbreviations

Aβ: Amyloid-β
AD: Alzheimer’s disease
ADAS-Cog: Alzheimer's Disease Assessment Scale–Cognitive subscale
AE: Adverse event
ApoE4: Apolipoprotein E4 allele
ARIA: Amyloid-related imaging abnormalities
ARIA-E: Amyloid-related imaging abnormalities corresponding to vasogenic edema
ARIA-H: Amyloid-related imaging abnormalities corresponding to microhemorrhages
AUC: Area under the curve
C: Control counts
ChEI: Cholinesterase inhibitor
CI: Confidence interval
CNS: Central nervous system
CSF: Cerebrospinal fluid
DMC: Data Monitoring Committee
DWI: Diffusion-weighted imaging
ECG: Electrocardiogram
FLAIR: Fluid-attenuated inversion recovery
ICH: Intracerebral hemorrhage
IFN-γ: Interferon-gamma
IgG: Immunoglobulin G
i.m.: Intramuscular
IQR: Interquartile range
LLOQ: Lower limit of quantification
mAb: Monoclonal antibody
MMSE: Mini-mental state examination
MRI: Magnetic resonance imaging
MSD: Meso Scale Discovery
PBMC: Peripheral blood mononuclear cells
R: Antigen counts
SAE: Serious adverse event
s.c.: Subcutaneous
SD: Standard deviation
SOC: System organ class
