# Supplementary material for: Long-term treatment with active Aβ immunotherapy with CAD106 in mild Alzheimer’s disease
Source: Alzheimers Res Ther. 2015 Apr 27;7(1):23. doi: 10.1186/s13195-015-0108-3 (PMC4410460; doi:10.1186/s13195-015-0108-3)
Supplement: Additional file 1: — List of independent ethics committees or institutional review boards. Independent ethics committee and institutional review board names and addresses for each study and each participating study center are given. [file 13195_2015_108_MOESM1_ESM.doc]

**List of Independent Ethics Committees or Institutional Review Boards**

**CCAD106A2201/2201E1; NCT00733863 and NCT00956410**

**France, Sweden, Switzerland and UK**

| **Center No.** | **Country** | **Ethics Committee or**  **Institutional Review Board** | **Address** |
| --- | --- | --- | --- |
| **0101** | France | CPP Sud ouest et Outre mer III | Bordeaux 33076  France |
| **0401** | Sweden | Regionala Etikprövningsnämnden i Stockholm | Stockholm 171 77  Sweden |
| **0501** | Switzerland | EKBB, Ethikkommission beider Basel | Basel CH-4056  Switzerland |
| **0601** | UK | Southampton and South West Hampshire Research Ethics Committee | Shirley Southampton, SO16 4RJ UK |

**CCAD106A2202/2202E1; NCT00795418 and NCT01023685**

**USA**

| **Center No.** | **Country** | **Ethics Committee or**  **Institutional Review Board** | **Address** |
| --- | --- | --- | --- |
| **00504** | USA | University of Texas Southwestern Medical Center | 5323 Harry Hines Blvd  Dallas, Texas 75390-8843 |
| **00505** | USA | Western Institutional Review Board | 3535 Seventh Ave.  SW Olympia,  Washington 98502-5010 |
| **00506** | USA | IUPUI/Clarian Institutional Review Board  (2202E1: Indiana University Institutional Review Board) | 620 Union Drive, Room 618  Indianapolis, IN 46202 |
| **00507; 00508; 00509; 00510; 00511** | USA | Quorum Review, Inc. | 1601 Fifth Avenue, Suite 1000  Seattle, WA 98101 |
